# Association of adrenal steroids with metabolomic profiles in patients with primary and endocrine hypertension

**DOI:** 10.3389/fendo.2024.1370525

**Published:** 2024-03-26

**Authors:** Robin Knuchel, Zoran Erlic, Sven Gruber, Laurence Amar, Casper K. Larsen, Anne-Paule Gimenez-Roqueplo, Paolo Mulatero, Martina Tetti, Alessio Pecori, Christina Pamporaki, Katharina Langton, Mirko Peitzsch, Filippo Ceccato, Aleksander Prejbisz, Andrzej Januszewicz, Christian Adolf, Hanna Remde, Livia Lenzini, Michael Dennedy, Jaap Deinum, Emily Jefferson, Anne Blanchard, Maria-Christina Zennaro, Graeme Eisenhofer, Felix Beuschlein

**Affiliations:** ^1^ Klinik für Endokrinologie, Diabetologie und Klinische Ernährung, UniversitätsSpital Zürich (USZ) und Universität Zürich (UZH), Zurich, Switzerland; ^2^ Université Paris Cité, Paris Cardiovascular Research Center (PARCC), L'Institut National de la Santé et de la Recherche Médicale (INSERM), Paris, France; ^3^ Département de Médecine Génomique des Tumeurs et des Cancers, Assistance Publique-Hôpitaux de Paris, Hôpital Européen Georges Pompidou, Paris, France; ^4^ Centre de référence en maladies rares de la surrénale, Hôpital Européen Georges Pompidou, Paris, France; ^5^ Division of Internal Medicine and Hypertension Unit, Department of Medical Sciences, University of Torino, Torino, Italy; ^6^ Medical Clinic III, University Hospital Carl Gustav Carus, Technische Universität (TU) Dresden, Dresden, Germany; ^7^ Institute for Clinical Chemistry and Laboratory Medicine, University Hospital and Medical Faculty Carl Gustav Carus, Technische Universität Dresden, Dresden, Germany; ^8^ Unita' Operativa Complessa (UOC) Endocrinologia, Dipartimento di Medicina DIMED, Azienda Ospedaliera-Università di Padova, Padua, Italy; ^9^ Department of Hypertension, National Institute of Cardiology, Warsaw, Poland; ^10^ Medizinische Klinik und Poliklinik IV, Klinikum der Universität München, Ludwig-Maximilians-Universität (LMU) München, Munich, Germany; ^11^ Department of Internal Medicine I, Division of Endocrinology and Diabetes, University Hospital, University of Würzburg, Würzburg, Germany; ^12^ Internal & Emergency Medicine Unit, Department of Medicine - DIMED, University of Padua, Padua, Italy; ^13^ The Discipline of Pharmacology and Therapeutics, School of Medicine, National University of Ireland, Galway, Ireland; ^14^ Department of Medicine, Section of Vascular Medicine, Radboud University Medical Center, Nijmegen, Netherlands; ^15^ Division of Population Health and Genomics, School of Medicine, University of Dundee, Dundee, United Kingdom; ^16^ Assistance Publique-Hôpitaux de Paris, Hôpital Européen Georges Pompidou, Centre d’Investigations Cliniques, Paris, France; ^17^ Assistance Publique-Hôpitaux de Paris, Hôpital Européen Georges Pompidou, Unité Hypertension artérielle, Paris, France; ^18^ The LOOP Zurich - Medical Research Center, Zurich, Switzerland

**Keywords:** metabolomics, adrenal steroids, endocrine hypertension, primary hypertension, pheochromocytoma, primary aldosteronism, Cushing’s syndrome

## Abstract

**Introduction:**

Endocrine hypertension (EHT) due to pheochromocytoma/paraganglioma (PPGL), Cushing’s syndrome (CS), or primary aldosteronism (PA) is linked to a variety of metabolic alterations and comorbidities. Accordingly, patients with EHT and primary hypertension (PHT) are characterized by distinct metabolic profiles. However, it remains unclear whether the metabolomic differences relate solely to the disease-defining hormonal parameters. Therefore, our objective was to study the association of disease defining hormonal excess and concomitant adrenal steroids with metabolomic alterations in patients with EHT.

**Methods:**

Retrospective European multicenter study of 263 patients (mean age 49 years, 50% females; 58 PHT, 69 PPGL, 37 CS, 99 PA) in whom targeted metabolomic and adrenal steroid profiling was available. The association of 13 adrenal steroids with differences in 79 metabolites between PPGL, CS, PA and PHT was examined after correction for age, sex, BMI, and presence of diabetes mellitus.

**Results:**

After adjustment for BMI and diabetes mellitus significant association between adrenal steroids and metabolites – 18 in PPGL, 15 in CS, and 23 in PA – were revealed. In PPGL, the majority of metabolite associations were linked to catecholamine excess, whereas in PA, only one metabolite was associated with aldosterone. In contrast, cortisone (16 metabolites), cortisol (6 metabolites), and DHEA (8 metabolites) had the highest number of associated metabolites in PA. In CS, 18-hydroxycortisol significantly influenced 5 metabolites, cortisol affected 4, and cortisone, 11-deoxycortisol, and DHEA each were linked to 3 metabolites.

**Discussions:**

Our study indicates cortisol, cortisone, and catecholamine excess are significantly associated with metabolomic variances in EHT versus PHT patients. Notably, catecholamine excess is key to PPGL’s metabolomic changes, whereas in PA, other non-defining adrenal steroids mainly account for metabolomic differences. In CS, cortisol, alongside other non-defining adrenal hormones, contributes to these differences, suggesting that metabolic disorders and cardiovascular morbidity in these conditions could also be affected by various adrenal steroids.

## 1 Introduction

Affecting more than 25% of the worldwide adult population, hypertension has become a global health challenge and is considered as one of the most important preventable risk factors for cardiovascular diseases (1, 2). Up to 90% of individuals with hypertension have no underlying disease and are therefore defined to have primary hypertension (PHT) (1, 2). However, among secondary and potentially curative causes, endocrine diseases such as pheochromocytoma and paraganglioma (PPGL), Cushing’s syndrome (CS) and primary aldosteronism (PA) together are common but often missed or diagnosed late (3–5). In addition to causing arterial hypertension, these disorders have been associated with several metabolic alterations, possibly explaining the higher cardiovascular risk of affected patients in comparison to those with PHT (6–9). Specifically, CS is well known to cause a detrimental form of metabolic syndrome including impairment of glucose metabolism and dyslipidemia among many other clinical manifestations (9). Similarly, PA has a higher prevalence of metabolic syndrome compared to patients with PHT (10), as well as a higher prevalence of obesity and some lipid abnormalities (11). Similarly, hormonally active PPGL result in metabolic alterations including impaired glucose and lipid metabolism (12). Improvements in these metabolic alterations have been demonstrated in patients with PPGL and CS (9, 12), and to a lesser extent in PA (11, 13), following surgical intervention. This further highlights a causal relationship between the endocrine disorder and the described metabolic perturbations. However, not all pathogenic mechanisms underlying these alterations have yet been clarified (11, 12).

Targeted metabolomics (TM) offers a recent approach to assess metabolic alterations in patients with endocrine-related hypertension (EHT) (14–17). The approach enables measurements of dozens to hundreds of low-weight metabolites within one biological sample, thereby providing a means to delineate pathogenic mechanisms (18, 19) and enable prognostic and diagnostic characterization/stratification (14, 20, 21). From a study in patients with PPGL (15), it is known that several observed metabolite alterations have commonalities with those in patients with metabolic disorders (such as diabetes mellitus (DM) and obesity). These observations provide possible insights into the pathogenesis of metabolism that might contribute to the increased cardiovascular risk associated with PPGL.

In a recent study that compared patients with PHT and EHT (PPGL, CS, PA), we identified differences in biogenic amines/amino acids (AA), glycerophospholipids (GP), and acylcarnitines (AC), that offer promise for diagnostic discrimination of patients with PHT and EHT (14). It might seem obvious that the disease-defining hormonal excess - catecholamines for PPGL, cortisol for CS and aldosterone for PA, respectively - would be the main cause of the observed metabolomic alterations for each form of EHT. However, in other studies of patients with PPGL and PA, some unexpected hypersecretion of adrenal steroid have been described, which might also contribute to the metabolic disturbances in these endocrine disorders (22, 23). Therefore, the objective of this study was to further delineate whether the disease-defining hormonal excess impacts metabolic differences alongside the effects of other secreted adrenal steroids. The results could further deepen the knowledge of pathogenic mechanisms linking metabolic alterations with increased cardiovascular risk in patients with EHT.

## 2 Materials and methods

### 2.1 Subjects

Data for the present analysis were derived from a previously published cohort of 294 patients with arterial hypertension enrolled at eleven centers of the ENSAT-HT consortium (http://www.ensat-ht.eu) (14). Enrolled participants were diagnosed according to the current guidelines either with PPGL, CS, PA or with PHT. For patients with PHT, additional secondary causes of arterial hypertension, particularly renal disease, pharmacological causes, obstructive sleep apnea syndrome, and those with low-renin hypertension, were meticulously ruled out and excluded. Furthermore, patients with severe comorbidities (e.g. active malignancy, chronic kidney injury or heart diseases) or pregnancy were excluded. The current study involved a subgroup of 263 patients who underwent steroid profiling. In addition to retrieved clinical data, body mass index (BMI) and presence/absence of DM were gathered from patients’ files. All participants provided written consent to participate in the study according to the protocol approved by the ethics committee of each center.

### 2.2 Laboratory testing

#### 2.2.1 Targeted metabolomics

Plasma samples were analyzed using the LC-ESI-MS/MS and FIA-ESI-MS/MS measurements of 188 metabolites. Full details about the method, including assay performance characteristics, exclusion of metabolites and normalization of data are described previously (14). For the purpose of this study, we included the measured values of those metabolites, which were significantly differing between patients with endocrine hypertension (PPGL, CS, PA) and primary hypertension in the mentioned study (14). Accordingly, in total, 79 metabolites and 18 metabolic indices (MI, containing metabolite ratios and sums) were included in this study. Details of the included metabolites and MI for each subgroup analysis (see below) are provided in **Supplementary Table 1.1**.

#### 2.2.2 Adrenal steroid profiling

Plasma steroid profiles included 15 different steroids (aldosterone, androstenedione, corticosterone, cortisol, cortisone, 11-deoxycorticosterone, 11-deoxycortisol, dehydroepiandrosterone (DHEA), dehydroepiandrosterone-sulfate (DHEAS), 17-hydroxyprogesterone, progesterone, testosterone, 21-deoxycortisol, 18-hydroxycortisol, 18-oxocortisol) that were analyzed using LC-MS/MS as described elsewhere (24).

#### 2.2.3 Plasma metanephrines

Measurements of plasma normetanephrine, metanephrine, 3-methoxytyramine and 3-O-methyldopa were performed using an LC-MS/MS. Further details and information on the procedure have been described previously (25, 26).

#### 2.2.4 Missing data estimation and data exclusion

We used a similar approach for exclusion and estimation for the adrenal steroids as we did for metabolite results previously (14, 15, 27, 28). In specific, we excluded those adrenal steroids with >10% of missing results (progesterone). The estimation of the values for those adrenal steroids with less than 10% of missing value was performed using the KNN approach (27, 28). In addition, for the purpose of this study we excluded the testosterone measurements, because of its strength relation to the sex. In total 13 out of 15 adrenal steroids were included in the further analyses.

### 2.3 Statistical analysis

Patients were categorized based on their diagnosis: PHT, PPGL, CS and PA. Baseline characteristics (age, sex) were compared across groups using the Pearson chi-squared test for sex and the Kruskal-Wallis test for age. Post-hoc analyses for comparisons between groups were performed using the Pearson chi-squared test for sex with a Bonferroni correction to account for multiple testing. The post-hoc analysis for age was conducted using Dunn-Bonferroni tests. For BMI comparisons, adjustments were made for age and sex using a one-way analysis of covariance (ANCOVA). When evaluating the presence of DM across groups, age and BMI were included as covariates. For these latter comparisons, BMI was logarithmically transformed due to non-normal distribution. A post-hoc Bonferroni correction for multiple testing was applied as well.

To assess differences in plasma steroid levels between the groups (PHT, PPGL, CS, and PA), a one-way ANCOVA was conducted with age and sex as covariates. Significance was determined using Bonferroni correction for multiple testing.

In a next step we focused on the 79 previously identified metabolomic differences and 18 different metabolic indices between the distinct clinical entities, namely PPGL, CS and PA, with PHT. In order to study the influence of the studied adrenal hormones, including also the leading (disease-identifying) hormone, we built multiple linear regression models separately for each comparison (PPGL-PHT, CS-PHT and PA-PHT) on the respective patients. In specific, each identified metabolite from the different comparisons (**Supplementary Table 1.1**) was considered as a dependent variable, whilst the leading hormonal excess and other adrenal steroid hormones as independent variables. Since metanephrines are a good diagnostic tool for PPGL but do not reflect the biological activity of catecholamines, we did not include these values in the regression models. Therefore, we introduced for the subgroup analysis with patients with PPGL (PPGL-PHT) a categorical variable reflecting only the presence or absence of catecholamine excess (CE). Because of the different distribution among subgroups, we included age and sex as variables in the models. In addition, we performed the same analyses including in addition BMI and DM in the model, for the subgroup of patients where these data were available (**Table 1**). After building our multiple regression models, we only included the results of the independent variables when the F-value of the regression models was statistically significant (p-value ≤ 0.05) (**Table 2**).

**Table 1:** General characteristics of the patients included in the study.

Diagnosis	PHT	PPGL	CS	PA	p-value
**Patients, n**	58	69	37	99	
**Females, n (%)**	19 (32.8)	38 (55.1)	34 (91.9)	42 (42.4)	<0.001^a^
**Age (years)**	44.8 (18.2-70.7)	53.8 (13.4-77.5)	51.2 (16.6-76.8)	48 (25.7-77.9)	<0.001^b^
**Patients including BMI & DM, n**	58	65	30	90	
**Females, n (%)**	19 (32.8)	36 (55.4)	27 (90.0)	39 (43.3)	<0.001^c^
**Age (years)**	44.8 (18.2-70.7)	53.7(13.4-77.5)	49.1 (16.6-73.3)	47.8 (25.7-77.9)	0.002 ^d^
**BMI (kg/m^2^)**	26.7 (19.6-40.6)	25.2 (16.0-34.3)	28.4 (20.9-39.5)	27.9 (18.0-41.0)	0.002 ^e^
**Diabetes mellitus (%)**	0 (0.0)	16 (24.6)	8 (26.7)	4 (4.4)	<0.001^f^

Numeric values (age, BMI) are presented as mean with minimum and maximum. Categorical data (patients, females, diabetes mellitus) are shown as absolute numbers with percentage within the group.

a Analyses in the distribution of sex in subgroups revealed a significant disparity among CS-PPGL, CS-PHT and CS-PA (all p<0.001).

b Comparison of age in subgroups showed a significant difference between median age between PA-PPGL (p=0.013) and PHT-PPGL (p=0.001).

c Analyses in the distribution of sex in subgroups revealed a significant disparity among CS-PPGL (p=0.005), CS-PHT (p<0.001) and CS-PA (p<0.001).

d Comparison of age in subgroups showed a significant difference between median age between PA-PPGL (p=0.018) and PHT-PPGL (p=0.002).

e Comparison of BMI in subgroups showed a significant difference between PPGL-PA (p=0.001) and PPGL-CS (p=0.005) after correction for age and sex.

f Comparison of diabetes mellitus occurrence in subgroups showed a significant difference between CS-PA (p=0.04), CS-PHT (p=0.003), PPGL-PA (p<0.001) and PPGL-PHT (p<0.001) after correction for age and BMI.

PA, primary aldosteronism; CS, Cushing’s syndrome; PPGL, pheochromocytoma and paraganglioma; PHT, primary hypertension; BMI, body mass index.

**Table 2:** List of studied metabolites and metabolic indices with the respective influencing adrenal steroids/catecholamines according to the subgroups.

	PPGL	CS	PA
Metabolite	Steroids & CE	Steroids	Steroids
Acylcarnitines			
C2	11DCS**		
C3-DC C4-OH	CE*, 11DCS*		
C7-DC			CST**, DHEAS**
C9		COL*, 11DCS*, 18OC*	18OHC*, 11DCS**, CST**, COL**, COS**, DHEA**
C10:1			11DCO*
C12:1			
C14:1	CE***, 11DCS***		
C14:2	COS*, 17OHP*		COS*, 11DCO***
C16			
C16:1	CE**, 11DCS**, 18OHC**		
C16:1-OH	11DCS*		
C18:1	CE**, 11DCS**, 18OHC**	11DCO**	Aldo*
C18:2	CE**, COL**, COS**, 11DCS*, 18OHC**	COL***, COS***	Aldo*, COS**
Amino Acids			
Arginine	CE**		
Aspartate	CE*, COL**, CST***	COL**	COL**, COS**, DHEA**, 17OHP**
Glutamate	COL**, 11DCO**, CST**	COL***, 18OHC***, DHEA***	Aldo*, CST*, COL***, COS***, DHEA**
Histidine	CE**, 21DC**		
Ornithine			CST**, COL**, COS**, DHEA**
Phenylalanine			Aldo*, 11DCS**, COS**, Ando**, DHEA**
Proline		18OHC**, 21DC***	
Serine			18OHC*, DHEA**, DHEAS**
Threonine			18OHC*, Ando**, DHEA**, DHEAS*
Biogenic Amines			
Spermidine	CE**, 17OHP**	11DCO*	Aldo**, 11DCO***
alpha-AAA	COL**, CST**, 18OC**		
Glycerophospholipids			
lysoPC a C14:0		COS**, 18OHC**, 17OHP**	
lysoPC a C16:0			COL*, COS*
lysoPC a C16:1		Ando**	
lysoPC a C17:0		Aldo*, 11DCS*	
lysoPC a C18:0	CST***		
lysoPC a C18:2	CE***, DHEAS***		
lysoPC C20:4	11DCS*		COS**
lysoPC a C24:0	CE**, COS**		
PC aa C32:1			
PC aa C32:2	CE*, DHEAS*		
PC aa C34:2			COL*, COS**, Ando**
PC aa C34:4	CE*, COS*, 21DC*		
PC aa C36:2	CE**, CST**		
PC aa C36:4	11DCS*		
PC aa C38:4	CST**, 11DCS*		
PC aa C38:6	CE*, 11DCO*, 18OC***, 17OHP**		
PC aa C40:1			COL*, COS*
PC aa C40:6	CE**, 11DCO*, 17OHP**		
PC aa C42:0			COL*, COS**, 11DCO*, DHEAS**
PC aa C42:1		11DCO**, 11DCS*, 18OHC**, 18OC**, DHEA**, 21DC***	COL*, COS**, 11DCO*, DHEAS**
PC aa C42:4			Aldo*
PC aa C42:5	CE***		
PC ae C32:1			
PC ae C32:2			11DCS*
PC ae C34:2	CE**, 18OHC**, 18OC**, DHEAS**		
PC ae C34:3	CE**, 11DCS*, 18OHC**, DHEAS***	COS***, Aldo**, 11DCO*, 11DCS*, 18OHC*, 18OC*	
PC ae C36:1		CST***, 11DCS**	
PC ae C36:3	COS*, 18OHC*, CE*		
PC ae C38:1			DHEA***
PC ae C40:3			11DCS**, COL*, COS*
PC ae C40:5			
PC ae C42:0	CE**, Ando*, 11DCO*	Aldo**, 11DCO**, 18OHC**, 18OC**, DHEA*, 17OHP**	18OHC*
PC ae C42:1			COL**, COS**
PC ae C42:2		11DCO*, 11DCS**	COL*, COS**, 11DCO*
PC ae C42:3			18OHC*, 18OC**, COL*, COS**, 11DCO*
PC ae C42:5			COL*, COS**, 11DCO*
PC ae C44:3			
PC ae C44:4		11DCO*, 11DCS*	
PC ae C44:5			COS***
PC ae C44:6			COL**, COS**, 11DCO*, 21DC*
Sphingolipids			
SM C16:1		CST***	
SM C18:0	CE**		
SM C18:1	CE**		Ando*
SM C20:2		COL***	
SM C24:1	CE**, 11DCO*		
SM(OH) C16:1		11DCS*	
**Monosaccharides**			
H1	CE*	Ando**, DHEA**	
Metabolic indices			
C2/C0			
Fischer Ratio	18OHC*		Aldo***, Ando***
CPT-I Ratio			Aldo**, COS**
Citrulline/Arginine	CE*		
Citrulline/Ornithine		COL**, COS**, 21DC**	CST*, COL**, COS**
Met-SO/Methionine	CE**		
Ornithine/Arginine	CE**, COS**	11DCO**	CST***, COL**, COS**, DHEA**
Putrescine/Ornithine			COL*, COS*
Spermidine/Putrescine	CE*, 17OHP**	18OHC*, DHEA*	Aldo*
Tyrosine/Phenylalanine	CE*, 11DCS***, DHEA*	DHEA*	
Total DMA/Arginine	CE**, COS**, DHEA**, 18OHC*		Aldo*
AAA			11DCS*, Ando*, DHEA*
BCAA			11DCS**, Ando**, DHEA**
Essential AA			11-DCS*, COS***, Ando**, DHEA**
Non-essential AA	21DC**		
Glucogenic AA		Ando*	
Total AA	21DC***		

Significant associated metabolites with steroids according to the subgroups (PPGL 127 patients without BMI/DM, 123 including BMI/DM and 57 metabolites/metabolic indices, CS 95 patients without BMI/DM, 88 including BMI/DM and 47 metabolites/metabolic indices, PA 157 patients without BMI/DM, 148 including BMI/DM and 57 metabolites/metabolic indices).

Highlighted fields represent not included metabolites in the subgroup analysis (dark grey) and metabolites where the regression model itself was not significant (lighter grey). Empty fields (white) represent missing predictor (adrenal steroid or catecholamine excess) in an otherwise significant regression model.

* Significantly associated metabolites in linear regression models only without BMI and DM in the model.

** Significantly associated metabolites in linear regression models with and without BMI and DM in the model.

*** Significantly associated metabolites in linear regression models only with BMI and DM in the model.

Abbreviations metabolites and metabolic indices: a, acyl; AA, amino acids; AAA, aromatic amino acids; aa, diacyl; ae, acyl-alkyl; BCAA, branched chain amino acids; CPT-I ratio, carnitine palmitoyltransferase I ratio; Cx:y shows the lipid chain composition where “x” is the number of carbons and “y” of double bonds. DMA, dimethylarginine; H1, sum of Hexoses (including Glucose); lysoPC, lysophosphatidylcholine; Met-SO, methionine sulfoxide; PC, phosphatidylcholine; SM, sphingomyelin.

Abbreviations steroids and clinical data: Aldo, Aldosterone; Ando, Androstenedione; BMI, body mass index; CE, catecholamine excess; COL, cortisol; COS, cortisone; CST, corticosterone; DHEA, dehydroepiandrosterone; DHEAS, dehydroepiandrosterone sulfate; DM, diabetes mellitus; CS, Cushing’s syndrome; PA, primary hyperaldosteronism; PHT, primary hypertension; PPGL, pheochromocytoma/paraganglioma; 11DCO, 11-deoxycortisol; 11DCS, 11-deoxycorticosterone; 17OHP, 17-hydroxyprogesterone; 18OC, 18-oxocortisol; 18OHC, 18-hydroxycortisol; 21DC, 21-deoxycortisol.

Since not normally distributed, data values of targeted metabolomics and steroid profiles were normalized using the GLOG-Transformation in the MetaboAnalyst platform prior to the analyses (28, 29). Statistical Analysis and figure building were performed using SPSS Statistics v27.0 (IBM).

## 3 Results

### 3.1 Cohort description

A total of 263 subjects were included in the study. In **Table 1**, general patient characteristics are provided. In short, mean age of the patients was 49.3 years (13.4-77.9) varying between the subgroups with significantly older patients in the PPGL group compared to PA and PHT. The distribution of patients by sex differs between the groups, with a predominance of female patients in the CS group compared to all others. In addition, there was a significant difference in BMI between the groups with lower BMI values in the PPGL groups compared to PA and PHT in the subgroup analysis. At last, a significant higher occurrence of DM was found in individuals with CS and PPGL compared to PA and PHT.

As expected, aldosterone and 18oxo-cortisol levels were higher in patients with PA compared to other groups (CS, PPGL and PHT) (**Supplementary Table 2**). In addition, 11-deoxycortisol levels were also significantly higher in patients with PA than in PHT. However, this was also the case in patients with CS and PPGL. Furthermore, in patients with CS, cortisol levels were higher than in PA and PHT, but not significantly higher than in patients with PPGL. DHEA and DHEAS levels were lower in CS compared to all other patients.

### 3.2 Associations of plasma steroids on metabolomic features within distinct disease groups: Regression analyses results

Details of the regression models, such as p-values and standardized beta regression coefficients, are provided in the **Supplementary Materials** (**Supplementary Tables 3.1, 3.2** for PPGL, **Supplementary Tables 3.3, 3.4** for CS and **Supplementary Tables 3.5, 3.6** for PA).

#### 3.2.1 Pheochromocytoma and Paraganglioma

**Figure 1:**
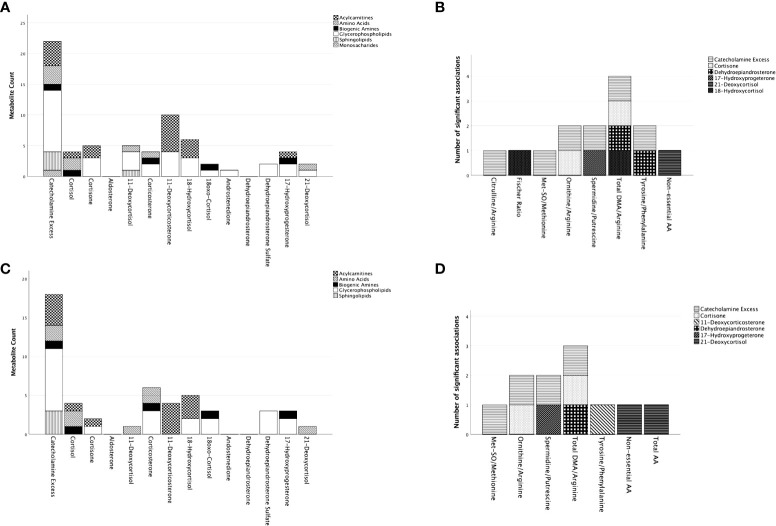
Summary of significant associations of adrenal steroids on the included metabolites and metabolic indices in patients with paraganglioma/pheochromocytoma. Represented are metabolites and metabolites indices for which the model in the multiple regression analyses resulted significant (**Supplementary Tables 3.1, 3.2**). In **(A)** (with adjustment for age/sex) and **(C)** (with adjustment for age/sex/BMI/DM) on the x-axis are represented the adrenal steroids with the total number of metabolites being significantly predicted for each steroid on the y-axis. The metabolites are represented by groups (acylcarnitines, amino acids, biogenic amines, glycerophospholipids, sphingolipids) defined by distinct pattering as specified in the figure legend. To note is that one metabolite might have significant associations with multiple adrenal steroids. **(B)** (with adjustment for age/sex) and **(D)** (with adjustment for age/sex/BMI/DM) represent the metabolite indices on the x-axis and the total number of adrenal steroid(s) significantly associated with the metabolic indices on the y-axis. Each adrenal steroid is represented by a different pattern as specified in the figure legend. AA, amino acids; DMA, dimethylarginine; Met-SO, sulfoxidized methionine.

Regression models were constructed for 57 metabolites/MI (**Supplementary Table 1.1**) for the cohort encompassing PPGL-PHT patients (**Table 2**; **Figure 1**).

In both models, CE had the most associations with metabolite levels with 22 of 42 metabolites in the model without BMI and DM, and 18 of 42 metabolites in the model including BMI and DM. The association was positive for all ACs, sphingolipids (SP), spermidine, and H1, while it was predominantly negative for AA and GP. Among all metabolites, GPs were the predominant one (10 out of 19 metabolites in the model without BMI and DM and 8 out of 19 metabolites in the model including BMI and DM). In both models, 11-deoxycorticosterone showed the most significant positive relationship with ACs (6 out of 12 metabolites in the model without BMI and DM and 4 out of 12 metabolites in the model including BMI and DM). Cortisol and corticosterone shared their association on AA with CE (2 out of 4 metabolites in both models) following aldosterone, DHEA, and androstenedione, which did not show any significant association.

#### 3.2.2 Cushing’s syndrome

**Figure 2:**
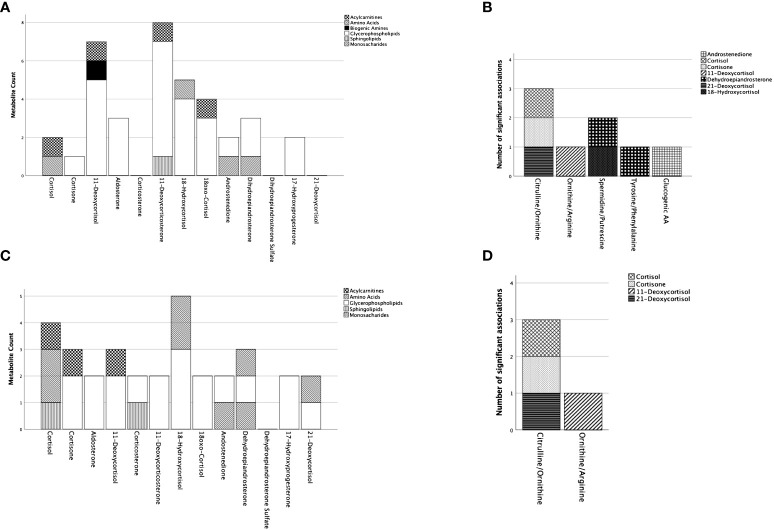
Summary of significant associations of adrenal steroids on the included metabolites and metabolic indices in patients with Cushing’s syndrome. Represented are metabolites and metabolites indices for which the model in the multiple regression analyses resulted significant (**Supplementary Tables 3.3, 3.4**). In **(A)** (with adjustment for age/sex) and **(C)** (with adjustment for age/sex/BMI/DM) on the x-axis are represented the adrenal steroids with the total number of metabolites being significantly predicted for each steroid on the y-axis. The metabolites are represented by groups (acylcarnitines, amino acids, biogenic amines, glycerophospholipids, sphingolipids) defined by distinct pattering as specified in the figure legend. To note is that one metabolite might have significant associations with multiple adrenal steroids. **(B)** (with adjustment for age/sex) and **(D)** (with adjustment for age/sex/BMI/DM) represent the metabolite indices on the x-axis and the total number of adrenal steroid(s) significantly associated with the metabolic indices on the y-axis. Each adrenal steroid is represented by a different pattern as specified in the figure legend. AA, amino acids.

Regression models were constructed for 47 metabolites/MI (**Supplementary Table 1.1**) for the cohort containing CS-PHT patients. (**Table 2**; **Figure 2**).

Cortisol had a negative association with C9 and citrulline/ornithine and a positive association with aspartate. Cortisone was only related to one metabolite, lysoPC acyl (a) C14:0. However, when BMI and DM were included in the linear regression models, the number of associations increased for cortisol (4 out of 37 metabolites) and cortisone (3 out of 37 metabolites). Specifically, C18:2 showed a significant relationship with both cortisol and cortisone. Additionally, glutamate and sphingomyelin (SM) C20:2 had positive relationships with cortisol, while PC acyl-alkyl (ae) C34:3 had a negative relationship with cortisone. Without including BMI and DM, 11-deoxycortisone (8 out of 37) and 11-deoxycortisol (7 out of 37) had the most significant associations with the metabolites. However, the number of their associations was less pronounced after the inclusion of BMI and DM in the model (11-deoxycortisone 2 out of 37, 11-deoxycortisol total 3 out of 37), whereas cortisol, 18-hydroxycortisol (5 out of 37), and DHEA (3 out of 37) had more associations with the metabolites. Moreover, monosaccharides showed associations with androstenedione and DHEA in both models (**Supplementary Figure 1**), while AA had associations with only cortisol (1 out of 6) and 18-hydroxycortisol (1 out of 6) without considering BMI and DM. After including BMI and DM, associations for AA were observed with cortisol (2 out of 6), 21-deoxycortisol (1 out of 6), 18-hydroxycortisol (2 out of 6), and DHEA (1 out of 6). Additionally, upon including BMI and DM, AC had associations with cortisol, cortisone, and 11-deoxycortisol (all 1 out of 10). GP mainly showed associations with 11-deoxycorticosterone (6 out of 13), then 11-deoxycortisol (5 out of 13), 18-hydroxycortisol (4 out of 13), and 18-oxocortisol (3 out of 13) before adding BMI and DM to the regression models. Ornithine, phenylalanine, and alanine did not have predictive associations with any of the studied adrenal steroids.

#### 3.2.3 Primary aldosteronism

**Figure 3:**
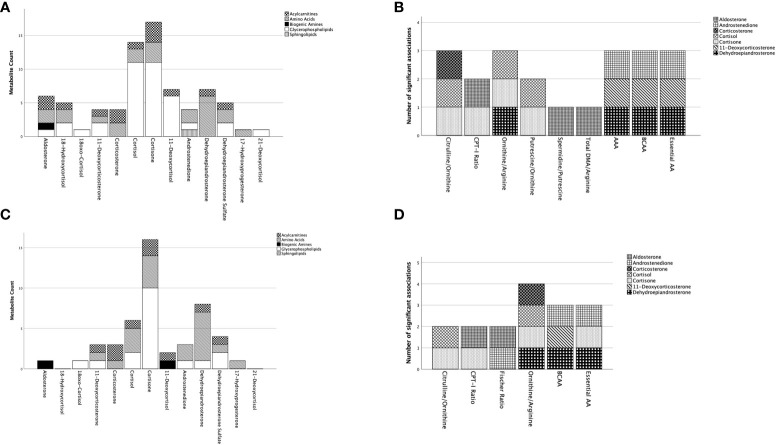
Summary of significant associations of adrenal steroids on the included metabolites and metabolic indices in patients with primary aldosteronism. Represented are metabolites and metabolites indices for which the model in the multiple regression analyses resulted significant (**Supplementary Tables 3.5, 3.6**). In **(A)** (with adjustment for age/sex) and **(C)** (with adjustment for age/sex/BMI/DM) on the x-axis are represented the adrenal steroids with the total number of metabolites being significantly predicted for each steroid on the y-axis. The metabolites are represented by groups (acylcarnitines, amino acids, biogenic amines, glycerophospholipids, sphingolipids) defined by distinct pattering as specified in the figure legend. To note is that one metabolite might have significant associations with multiple adrenal steroids. **(B)** (with adjustment for age/sex) and **(D)** (with adjustment for age/sex/BMI/DM) represent the metabolite indices on the x-axis and the total number of adrenal steroid(s) significantly associated with the metabolic indices on the y-axis. Each adrenal steroid is represented by a different pattern as specified in the figure legend. AA, amino acids; AAA, aromatic amino acids; BCAA, branched chain amino acids; CPT-I ratio, carnitine palmitoyltransferase I ratio; DMA, dimethylarginine.

Multiple linear regression analyses were performed for each of the 57 metabolites/MI (**Supplementary Table 1.1**) studied in the cohort consisting of patients with PA and PHT (**Table 2**; **Figure 3**).

Among the 57 metabolites/MI, only nine were associated with aldosterone levels. Among these, C18:2, spermidine, and phosphatidylcholine (PC) diacyl (aa) C42:4, as well as CPT-I ratio, spermidine/putrescine, and total DMA/arginine, were positively associated with aldosterone. However, after adding BMI and DM to the model, these associations with aldosterone were no longer significant, except for spermidine, CPT-I ratio, and the Fisher-ratio (not resulted significant in the former model).

In contrast, a higher number of metabolites (14 in total) were predicted by cortisol, including a negative association with short-chain AC, two positively associated AA, as well as 11 GP. Similarly, 17 metabolites were predicted by cortisone, containing two negatively and one positively associated AC, three negatively associated AA, as well as 11 negatively associated GP. Interestingly, cortisone and cortisol had the most associations with GP (11 out of 27) and DHEA on AA (6 out of 7). After including BMI and diabetes in the model, the significance of glucocorticoids persisted. However, cortisol had fewer associations with GP (2 out of 27), whereas cortisone remained associated with almost the same number of GP (10 out of 27), AA (4 out of 7), and AC (2 out of 9). Additionally, the significance of the association between DHEA and AA did not change. The other parameters (18-hydroxycortisol, 18oxo-cortisol, 11-deoxycortisone, corticosterone, 11-deoxycortisol, androstenedione, DHEAS, 17-hydroxyprogesterone) showed only a few associations.

## 4 Discussion

From earlier studies (23), it has been appreciated that hormonal alterations in patients with PPGL, CS and PA are not only characterized by excessive secretion of the disease-defining adrenal hormones but also by concurrent hormonal alterations that vary significantly between the disorders. In this study, we further identified associations between these hormonal changes and the metabolomic differences previously reported (14), at times exceeding the associations seen with the disease-defining hormonal excess.

Considering the whole cohort of hypertensive patients, we identified a high number of associations between distinct adrenal steroids on the acylcarnitines and glycerophospholipids. From the literature, it is known that long-chain acylcarnitines are associated with cardiac morbidities such as arrhythmic disorders (6), dilated cardiomyopathy as well as heart failure (30). In addition, long-chain acylcarnitines have been associated with insulin resistance and DM type 2 (31). It is of interest that all these clinical features are more prominent in patients with endocrine hypertension in comparison to those with PHT (6–9). Interestingly, in addition to the disease defining catecholamine excess in patients with PPGL, and cortisol excess in patients with CS, we found that cortisone, 11-deoxycortisol and 11-deoxycorticosterone exhibited a significant association with long-chain acylcarnitines.

Another crucial aspect to consider across all three EHT entities is the increased catabolic state and loss of skeletal muscle (32–34), leading to increased protein turnover. This is reflected in higher levels of aspartate and glutamate within the metabolomic profile of patients affected by EHT. We also observed an association between cortisol and both amino acids in all EHT subgroups, in concomitance with the association of aspartate with catecholamine excess in PPGL and glutamate with aldosterone in the PA patients. Surprisingly, DHEA was associated with both metabolites in PA and with glutamate in CS subgroup analysis. Notably, considering the potential role of aspartate and glutamate in the pathogenesis of glucose homeostasis disorders (35), it is intriguing to observe that lower levels of DHEA, which tend to be present in patients with CS and PA compared to patients with PHT, were associated with higher levels of these two amino acids.

In addition, considering the measured metabolic indices as surrogate markers of specific metabolic pathways, we identified a large number of associations with urea cycle activity (represented by citrulline/ornithine, ornithine/arginine), activity of protein arginine methyl transferases (represented by total DMA/arginine), and CPT-I activity. Similarly to the above-described metabolites, available data reveal the importance of arginase and protein arginine methyl transferases in the pathogenesis of hypertension, obesity, insulin resistance and diabetes mellitus (36–41).

In patients with PPGL the most significant association on the observed metabolomic differences compared to patients with PHT was related the catecholamine excess itself. Specifically, metabolomic markers associated with higher cardiovascular risk and dysglycemia (long-chain acylcarnitines, glycerophospholipids, arginine) as well as metabolomic indices, including PRMT (total DMA/Arginine) and arginase (ornithine/arginine) activity (6, 30, 31, 36–43) were mainly associated with the extent of catecholamine excess. Nevertheless, adrenal steroids, such as 11-deoxycortisone, and after adjustment for BMI and DM, corticosterone, 18-hydroxycortisol, and cortisol, also showed associations with these metabolic markers. Interestingly, 11-deoxycortisone had most of the positive associations with long-chain acylcarnitines followed by 18-hydroxycortisol, adding some additional impact on the increased cardiovascular risk observed in the patients with PPGL (7). However, a CE-independent metabolomic difference regarded the alpha-aminoadipic acid (AAA). AAA is a product of the lysine breakdown pathway, and one study hypothesized that AAA might be part of the carbonyl stress pathway seen in diabetes (44). Furthermore, higher levels of AAA have been linked with diabetes mellitus and disturbed glucose metabolism (45), which are more often seen in patients with PPGL (12).

In patients with CS, it was evident that the metabolomic traits, specifically GP (e.g., PC aa C42:1, PC ae C42:2, PC ae C44:4), were mainly associated with 11-deoxycorticosterone, 18-hydroxycortisol (positively), and 11-deoxycortisol (negatively), rather than cortisol itself before adjusting for DM and BMI. However, this changed after adjustment for these features, where, in addition to 11-deoxcycortisol, cortisol was mainly associated with the metabolomic profile. It is therefore tempting to speculate that, in addition to the well-known impact of cortisol on insulin resistance (9), mineralocorticoids may contribute to the respective metabolomic pattern (GP), related to insulin resistance (46, 47). The significant association with the low arginine level, potentially due to decreased urea cycle activity and heightened arginase activity, also involves 21-deoxycortisol and 11-deoxycortisol. This relationship further relates to the metabolic syndrome phenotype and increased cardiovascular risk (34, 41, 46). One particularly intriguing discovery is the notable association between glucose levels (indicated by monosaccharides) and DHEA, which is significantly reduced in patients with CS within our cohort. Lower DHEA is primarily observed in patients with adrenal-based, and thus ACTH-independent CS, which are mainly represented in our cohort (data has not been presented). This relationship is noteworthy, as a higher prevalence of diabetes mellitus is observed among patients with ACTH-dependent CS, which corresponds to elevated DHEAS levels when compared to those with ACTH-independent CS (47).

Intriguingly, in patients with PA, the analysis did not suggest aldosterone as the hormone with most associations with the metabolomic differences, but rather cortisone followed by cortisol and DHEA. Specifically, a strong association was observed between cortisone (negative correlation) and cortisol (positive correlation) with several GP (e.g., lyso PC a C16:0, PC aa C34:2, PC aa C42:1, PC ae C42:1, PC ae C44:6) before accounting for BMI and DM in patients with PA. Recent studies have shown a significant association between several GP and SP and insulin resistance/metabolic syndrome (46, 48, 49), which are phenotypic features observed in patients with PA (10, 11). Additionally, lyso PCs increase oxidative stress and regional vascular inflammation, and higher circulating levels of lyso PCs are associated with early coronary atherosclerosis leading to a higher cardiovascular risk (50, 51), which has been observed in patients with PA compared to PHT (3). Therefore, the metabolome traits distinguishing PA from PHT and associated with glucose metabolism alteration and cardiovascular morbidity are mainly associated with the concomitantly secreted adrenal steroids, rather than aldosterone itself. In line with the metabolomic traits, the MI in patients with PA, such as citrulline/ornithine and ornithine/arginine, which are associated with increased cardiovascular risk and phenotypic features of metabolic syndrome, were mainly associated with cortisol, cortisone, corticosterone, and DHEA in our regression analysis. Based on these observations, it can be speculated that the poorer cardiovascular outcomes in patients with PA treated pharmacologically by inhibiting aldosterone activity compared to surgery (52) might be related to the unopposed activity of the other adrenal steroids.

Overall, these findings provide indirect evidence that various adrenal hormones are linked to alterations in specific metabolic pathways that may also influence cardiovascular and metabolic risk.

The current study has several strengths, including a multicentric approach, a well-characterized patient cohort with arterial hypertension, and comprehensive endocrine assessments for diagnosis. The study protocol included standardized blood sample collection, minimizing the impact of external factors, and all measurements were performed on blood specimens obtained at the same time point.

There are, however, some limitations to our study: While the diagnosis of endocrine hypertension was made using standard laboratory screening tests in line with the guidelines at the study’s inception, no subsequent data were available to verify the initial diagnosis. Particularly concerning the diagnosis of PA, recent findings indicate potential over-diagnosis based on current cut-offs, dependent on the assay used (53, 54). Consequently, we cannot rule out potential misclassification of some cases in our cohort. Moreover, due to the study’s design, the modest patient count, and its retrospective nature, there might be an overestimation in our results, suggesting associations rather than definitive causations. The statistical approach, utilizing linear regression, encompassed multiple predictors. This increases the likelihood of collinearity or confounding, especially compared to models with fewer parameters. Unfortunately, data on substance abuse (nicotine, alcohol) weren’t accessible, and only a limited array of clinical data was on hand. The sample sizes for certain subgroup disorders, notably in the CS group, were relatively minimal. Therefore, our study’s findings should serve as a foundational reference for subsequent research in a prospective environment, emphasizing the accumulation of broader clinical information from a more extensive population. Data post-treatment would further validate the nature of the relationship. Additionally, any speculative interpretation of the observed links requires extensive background validation and mechanistic studies to ascertain causality. Addressing this point, it’s important to recognize that, although distinct metabolic patterns have been observed, certain associated adrenal steroid concentrations didn’t showcase significant variance between the groups. This highlights a possible context-specific exposure yet to be identified.

In conclusion, our study suggests a significant impact of cortisol, cortisone, and catecholamine excess on the metabolomic differences observed in patients with EHT compared to PHT. Surprisingly, apart from patients with PPGL, where the catecholamine excess played a major role in the metabolomic changes, the majority of metabolomic differences observed in patients with PA were associated with non-disease defining hormonal excess represented by other adrenal steroids from the studied panel. In CS, cortisol was also not the leading adrenal steroid but was associated with metabolomic differences along with other non-disease defining adrenal hormones. These findings suggest that the metabolic disorders and increased cardiovascular morbidity in these patients may be influenced by other adrenal steroids as well.

## Data availability statement

The original contributions presented in the study are included in the article/**Supplementary Material**. Further inquiries can be directed to the corresponding author.

## Ethics statement

The studies involving humans were approved by Kantonale Ethikkomission Zürich. The studies were conducted in accordance with the local legislation and institutional requirements. The participants provided their written informed consent to participate in this study.

## Author contributions

RK: Data curation, Formal analysis, Investigation, Methodology, Visualization, Writing – original draft, Writing – review & editing. ZE: Conceptualization, Data curation, Formal analysis, Funding acquisition, Investigation, Methodology, Project administration, Resources, Supervision, Validation, Visualization, Writing – original draft, Writing – review & editing. SG: Data curation, Investigation, Resources, Writing – review & editing. LA: Data curation, Investigation, Resources, Writing – review & editing. CL: Data curation, Investigation, Resources, Writing – review & editing. A-PG-R: Data curation, Investigation, Resources, Writing – review & editing. PM: Data curation, Investigation, Resources, Writing – review & editing. MT: Data curation, Investigation, Resources, Writing – review & editing. APe: Data curation, Investigation, Resources, Writing – review & editing. CP: Data curation, Investigation, Resources, Writing – review & editing. KL: Data curation, Investigation, Resources, Writing – review & editing. MP: Data curation, Formal analysis, Investigation, Methodology, Resources, Supervision, Writing – review & editing. FC: Data curation, Investigation, Resources, Writing – review & editing. APr: Data curation, Investigation, Resources, Writing – review & editing. AJ: Data curation, Investigation, Resources, Writing – review & editing. CA: Data curation, Investigation, Resources, Writing – review & editing. HR: Data curation, Investigation, Resources, Writing – review & editing. LL: Data curation, Investigation, Resources, Writing – review & editing. MD: Data curation, Investigation, Resources, Writing – review & editing. JD: Data curation, Investigation, Resources, Writing – review & editing. EJ: Data curation, Investigation, Resources, Writing – review & editing. AB: Data curation, Investigation, Resources, Writing – review & editing. M-CZ: Data curation, Investigation, Project administration, Resources, Supervision, Writing – review & editing. GE: Conceptualization, Data curation, Funding acquisition, Investigation, Methodology, Project administration, Resources, Supervision, Validation, Writing – original draft, Writing – review & editing. FB: Conceptualization, Data curation, Funding acquisition, Investigation, Methodology, Project administration, Resources, Supervision, Validation, Writing – original draft, Writing – review & editing.
