# Supplementary material for: Association of adrenal steroids with metabolomic profiles in patients with primary and endocrine hypertension
Source: Front Endocrinol (Lausanne). 2024 Mar 26;15:1370525. doi: 10.3389/fendo.2024.1370525 (PMC11002274; doi:10.3389/fendo.2024.1370525)
Supplement: Supplementary file 1 [file DataSheet_1.docx]

Supplementary Material

# Supplementary Figures and Tables

## Supplementary Figure 1: Venogram of Sum of Hexose (H1)

Represented as a venogram with the corresponding diagnosis as a circle (PA, PPGL, CS) are the results of significant associations of different steroids as well as catecholamine excess and the sum of hexose (H1). An Asterix indicates significance only prior to body mass index and diabetes mellitus correction.

**Abbreviations**: Ando, Androstenedione; CE, catecholamine excess; CS, Cushing’s syndrome; DHEA, dehydroepiandrosterone; PA, primary hyperaldosteronism; PPGL, pheochromocytoma/paraganglioma

## Supplementary Tables

### Supplementary Table 1.1: Complete list of included metabolites in the different subgroup analyses

### Supplementary Table 1.2: List of adrenal steroids and metanephrines

### Supplementary Table 2: Differences in adrenal steroid levels between different clinical entities

### Supplementary Table 3.1: Complete list of significant regression models of each included metabolite in the subgroup containing patients with paraganglioma/pheochromocytoma and primary hypertension.

### Supplementary Table 3.2: Supplemental Table 3.2 Complete list of significant regression models of each included metabolite in the subgroup containing patients with paraganglioma/pheochromocytoma and primary hypertension including body mass index and diabetes mellitus.

### Supplementary Table 3.3: Complete list of significant regression models of each included metabolite in the subgroup containing patients with Cushing’s syndrome and primary hypertension.

### Supplementary Table 3.4: Complete list of significant regression models of each included metabolite in the subgroup containing patients with Cushing’s syndrome and primary hypertension including body mass index and diabetes mellitus.

### Supplementary Table 3.5: Complete list of significant regression models of each included metabolite in the subgroup containing patients with primary aldosteronism and primary hypertension.

### Supplementary Table 3.6: Complete list of significant regression models of each included metabolite in the subgroup containing patients with primary aldosteronism and primary hypertension including body mass index and diabetes mellitus.
